# Effects of chronic leptin infusion on subsequent body weight and composition in mice: Can body weight set point be reset?^a^^a^

**DOI:** 10.1016/j.molmet.2014.02.003

**Published:** 2014-03-05

**Authors:** Y. Ravussin, C.A. LeDuc, K. Watanabe, B.R. Mueller, A. Skowronski, M. Rosenbaum, R.L. Leibel

**Affiliations:** Department of Pediatrics, Division of Molecular Genetics, Columbia University, College of Physicians and Surgeons, 630 West 168th Street, New York, NY 10032, USA

**Keywords:** Leptin, Body weight “set-point”, Obesity, Leptin resistance

## Abstract

Circulating leptin concentrations correlate with fat mass and signal the status of somatic energy stores to the brain. Previous studies suggest that diet-induced elevations of body weight increase body weight “set-point”. To assess whether chronic hyperleptinemia is responsible for this shift in defended body weight, we elevated circulating leptin concentrations in lean mice to those comparable to diet-induced obese mice for eighteen weeks. We hypothesized that following cessation of leptin infusion, a higher body weight would be defended. Compared to saline-infused controls, leptin-infused mice had elevated circulating leptin concentrations, gained less weight, yet had similar metabolic rates. Following cessation of leptin administration, leptin-infused mice gained some weight yet plateaued at 5–10% below controls. These results suggest that, unlike mice rendered hyperleptinemic by diet-induced weight gain, leptin-infused mice do not subsequently “defend” a higher body weight, suggesting that hyperleptinemia *per se* does not mimic the CNS consequences of chronic weight gain.

## 1 Introduction

In weight-stable individuals, circulating leptin concentrations are directly proportional to fat mass [1]. Following weight-loss, the decrease in circulating leptin concentration due to reduced fat mass provides one of the signals that induce a CNS-mediated decrease in energy expenditure and increase in hunger in lean and obese humans [2–4] and rodents [5]. Restoration of leptin concentrations to pre-weight loss levels abrogates some of these metabolic, neuroendocrine, autonomic, and behavioral responses [3,6]. In contrast, 48-h fasted male mice show starvation-induced changes in gonadal, adrenal, and thyroid axes that are all leptin-reversible without significant effect on body weight or on re-feeding after starvation [7]. Taken together, these findings indicate that circulating leptin concentration is a major afferent signal of overall energy availability, and that the hypometabolic phenotype of weight-reduced individuals is the result – at least in part – of a state of perceived relative leptin insufficiency [8].

In contrast to the potent effect of leptin administration to humans or rodents with leptin insufficiency (weight-reduced, fasted, or congenitally leptin deficient), administration of physiological or supraphysiological doses of leptin to rodents or humans at usual or increased body weight has little to no effect on energy expenditure or food intake [3,9–11]. Such data suggest that leptin-sensing circuitry in the CNS is “designed” to be inherently more responsive to a deficit of ambient leptin rather than an excess [7,12]. Evolutionary arguments for such “asymmetric” regulatory responses to changes in body fat have been proposed [13]. Determination of the “threshold” (minimum signal regarding fat mass) below which these responses are invoked is determined by genetic and developmental factors [13].

An important question is whether this threshold can be reset by the environment; i.e., whether sustained maintenance of a body weight higher (or lower) than that “encoded” by genetic and early developmental factors, could permanently alter the level of body fat “defended” by an individual. Such malleability would have major implications for efforts to prevent and treat obesity. Studies of humans and rodents sustaining weight loss indicate that the hypometabolic state evident shortly after weight loss persists over long periods of time if not indefinitely [14,15]. In contrast, prolonged elevation of body weight results in defense of an elevated level of body fatness compared to never-obese control mice [5]. Elevations in the hypothalamus of ambient free fatty acids [16] or cytokines (e.g. IL6) [17], and impairments of molecular stress responses in the endoplasmic reticulum [18], can reduce acute and chronic leptin signaling, accounting perhaps for the persistence of high levels of body fat despite proportionate elevations of circulating leptin concentrations in DIO mice [12]. Whether these various desensitization processes occur by a shared mechanism is unknown [19], and whether such desensitization is mechanistically related to the apparent “defense” of a higher body weight in mice chronically maintained at higher body weight by feeding of a HFD, is not clear.

In the present study we sought to isolate the possible effects of high ambient leptin *per se* on the body weight that is defended, i.e., to experimentally disarticulate hyperleptinemia from the many changes in metabolic and neuroendocrine systems that occur as the result of increased body fatness and of the high fat content of the diet used to attain this level of fatness. We examined the effects of 18 weeks of continuous exogenous leptin infusion on the metabolic responses of C57BL/6J mice. By infusing recombinant murine leptin in lean mice consuming a low-fat diet – to achieve circulating concentrations that mimicked those of age-matched diet-induced obese (DIO) mice – we sought to create a mouse model of elevated circulating leptin concentrations without the metabolic “confounds” of diet-induced obesity (e.g. elevated FFA and glucose, insulin insensitivity, fatty liver, etc.). Our hypothesis was that a chronic elevation in leptin concentrations would result in a permanent elevation in the minimum level of defended body fat.

## 2 Materials and methods

### 2.1 Animals

48 C57BL/6J male 6 week-old mice were obtained from Jackson Laboratory (Bar Harbor, ME). Upon receipt, animals were housed 4 per cage in plastic pens with wood chip bedding in a pathogen-free barrier facility maintained at 22–24 °C with a 12-h dark–light cycle (lights on at 07:00 h). *Ad libitum* access to a low fat diet (LFD: Research Diets, Inc. D12450Bi, 10% kcal from fat) and water were provided during the entire experiment unless otherwise specified. Body weight and body composition were determined every 14 days unless otherwise specified.

The protocol was approved by the Columbia University Institutional Animal Care and Use Committee.

### 2.2 Study design overview

**Figure 1:**
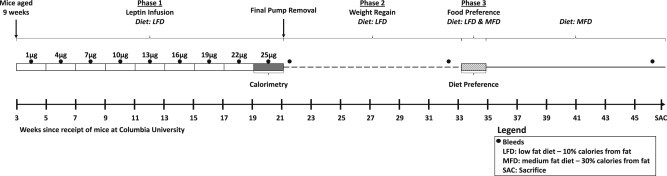
Experimental Timeline. Experimental timeline of all three phases of the study. Following a 3 week acclimatization period at Columbia University, mice were nine weeks old at the start of the experiment. Mini-pumps containing increasing quantities of leptin were surgically replaced every two weeks during an 18-week infusion period (Phase 1). Blood was obtained by retro-orbital bleed following a 4-h fast eight days after each mini-pump switch during the 18 weeks of Phase 1 as well as well as 5, 41, and 118 days following removal of the final pump (see black circles).

**Figure 2:**
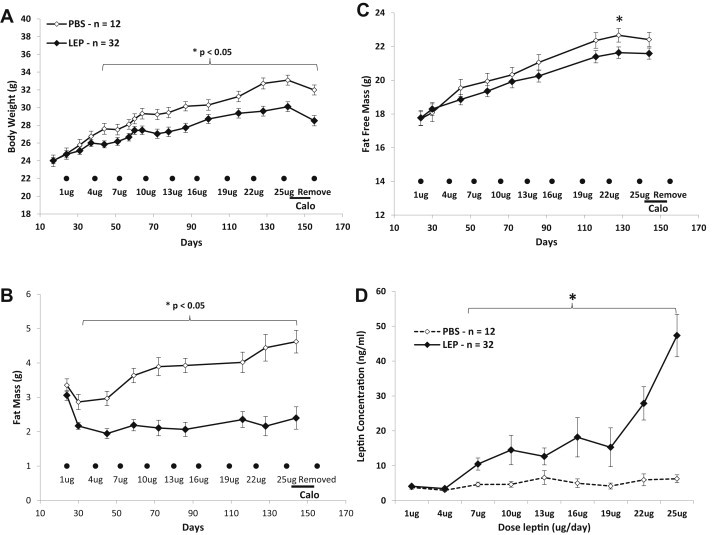
Body weight, body composition, and circulating leptin concentration during Leptin Infusion phase **(A–C)** Mean (±sem) body weight **(A)** fat mass **(B)** and fat-free mass **(C)** of leptin- (LEP mice; *n* = 32; black diamond) or PBS- (PBS mice; *n* = 12; open diamonds) infused mice. Black circles represent mini-pump replacement surgeries (doses of leptin are noted below each circle in μg/day). Black line on bottom right represents 72 h indirect calorimetry measures for all mice. **(D)** Mean (±sem) serum leptin concentrations (ng/ml) at increasing leptin infusion rates (μg/day) of LEP and PBS groups collected from four-hour fasting serum obtained 7 days following mini-pump replacement surgery. * *p* < 0.05.

**Figure 3:**
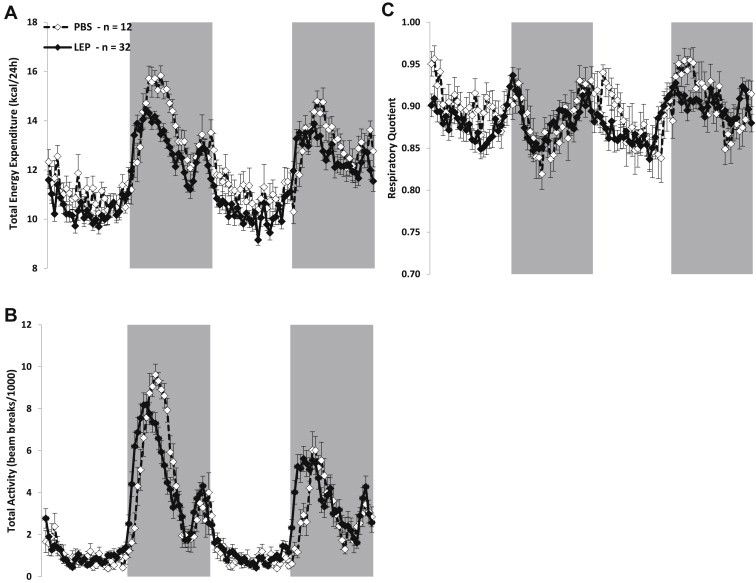
Energy expenditure, ambulatory activity, and respiratory during Leptin Infusion phase. Mean (±sem) non-adjusted (for body weight or composition) total energy expenditure **(A)**, total activity **(B)**, and respiratory quotient **(C)** of leptin- (LEP mice; *n* = 32; black diamond) or PBS- (PBS mice; *n* = 12; open diamond) infused mice. Gray shading represents lights off period. Mice spent 72 h in calorimeter; data was collected during the last 48 h for all three figures.

**Figure 4:**
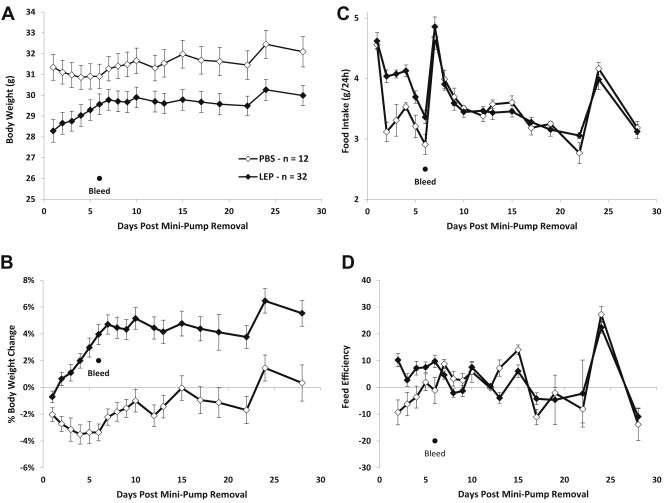
Body weight, food intake, and metabolic efficiency during Weight Regain phase. Mean (±sem) body weight **(A)** percent body weight change **(B)** 24-h food intake of low fat (10% kcal from fat) control diet **(C)** and 24-h feed efficiency (calculated by dividing 24-h weight change (g) by 24-h food intake (g)) **(D)** of leptin- (LEP mice; *n* = 32; black diamond) or PBS- (PBS mice; *n* = 12; open diamond) infused mice 30 days following removal of the terminal mini-pump.

**Figure 5:**
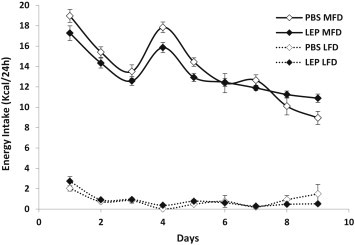
Food intake during Diet Preference phase. Mean (±sem) kcal/24 h consumed of either low fat diet (LFD: 10% kcal from fat: dashed lines) or medium fat diet (MFD: 30% kcal from fat: solid lines) for either leptin- (LEP mice; *n* = 32; black diamond) or PBS- (PBS mice; *n* = 12; open diamond) infused mice.

There were 3 phases to this study (see Figure 1 for overview): 1. Leptin Infusion (Figures 2 and 3); 2. Weight Regain (Figure 4); and 3. Food Preference (Figure 5). All animals participated in all phases. After a 3-week acclimatization period, cages were stratified based upon the total weight of the 4 mice occupying each cage. Cages were arranged in triplicates in ascending order of total body weight, with cages 1 and 2 being assigned to leptin (LEP; *n* = 36) and cage 3 of each triad assigned to PBS (*n* = 12). Mean body weights for the animals assigned to PBS (*n* = 12) and LEP (*n* = 36) were 24 ± 0.6 g and 24 ± 0.3 g, respectively (Figure 2). Mini-pumps (Alzet, Cupertino, CA, models 1002 or 2002) were implanted in all mice. The LEP mice received recombinant murine leptin dissolved in PBS (7.9 pH: source = Dr. A.F. Parlow; National hormone & peptide program); the PBS mice received vehicle. Pumps were surgically replaced every two weeks during an 18-week infusion period (**Leptin Infusion phase**). Mice were placed in metabolic chambers to assess their energy expenditure 6 days after the implantation of the final mini-pump (25 μg/day: days 144–151; Figures 1, 2A and 3A–C). Mini-pumps were subsequently removed (day 155; Figures 1 and 2A), and food intake and body weight were monitored for the following 25 days (**Weight Regain phase**: Figures 1 and 4A–D). Forty-eight days after cessation of leptin or saline infusion (during which animals had *ad-libitum* access to the LFD), a diet preference test was conducted (**Diet Preference phase**: Figures 1 and 5). Mice were individually housed and given simultaneous access to both a LFD (10% kcal from fat) and a medium fat diet (MFD: 30% kcal from fat: Research Diets, Inc. D09082404i) placed on the floor of the cage. Each food source was weighed daily for 10 days. Following 10 days of access to both LFD and MFD with no significant differences in diet preference between the LEP and PBS mice (Figure 5), mice were given *ad-libitum* access only to the MFD for the remainder of the experiment (a total of 60 days). Four LEP mice died during surgery and all data from those mice were excluded from the final analyses.

### 2.3 Leptin Infusion phase

#### 2.3.1 Mini-pumps

Nine mini-pump implantations (at 2-week intervals) were performed in each animal over the 18-week infusion period. The first 6 mini-pumps used were Alzet model 1002; the last 3 were model 2002 (Alzet; Cupertino, CA) that holds twice the volume (200 μl) of a model 1002, and was required to increase the leptin delivered without exceeding the maximum solubility of the leptin used in this experiment (leptin maximum concentration = 1 μg/μl). Animals were anesthetized with inhaled isoflurane (2–3.5%) in oxygen, and mini-pumps placed in a dorsal subcutaneous pouch. Shaved skin was prepped with betadine and alcohol washes, and a 1.5–2 cm incision caudal to the interscapular region was made, avoiding the interscapular brown adipose tissue depot. The mini-pump was positioned with the orifice facing caudally. Wounds were closed using EZ Clip Wound Closing Kit – 9 mm (Stoelting; Wood Dale, IL) and clips were removed 6 days after pump insertion and 1 day before body composition measurements. The metal flow moderator was replaced with PEEK medical microtubing (Durect Corporation, Cupertino, CA) to enable use of the time-domain-NMR for body composition analysis. To assess the effect of this retrofit on flow rate from the pump, we did not change out the metal flow modulator on mini-pump change 7. This step accounts for the absence of body composition measures for that period (16 μg/day; days 94–109; Figures 2B and C). Leptin dosing was based on pilot studies in which we correlated rates of infusion with intercurrent circulating serum concentrations of leptin. Using regression analyses of fat mass and serum leptin made earlier [5], our goal was to elevate circulating leptin concentrations in non-obese LFD-fed mice to those analogous of age-matched diet-induced obese mouse. The lowest dose administered was 1 μg/day/mouse (42 ng/h) and the highest dose was 25 μg/day/mouse (1042 ng/h); 3 μg/day increments were added at each mini-pump switch time point (i.e. 4 μg/day, 7 μg/day, etc.). Four-hour fasting blood was obtained by retro-orbital bleeding 7 days after each mini-pump implantation.

#### 2.3.2 Body weight and body composition

Body weight (BW) was measured (±0.1 g) weekly using an Ohaus Scout Pro 200 g scale (Nänikon Switzerland, between 07:45–08:15 h). Body composition (fat mass: FM, fat-free mass: FFM, & extracellular fluid) were measured on a mouse carcass-calibrated [20] time-domain-NMR (Minispec Analyst AD; Bruker Optics, Silberstreifen, Germany) every 2 weeks (1 week after previous mini-pump implantation and one day before retro-orbital bleeding).

#### 2.3.3 Indirect calorimetry

Energy expenditure was measured with a LabMaster-CaloSys-Calorimetry System (TSE Systems, Bad Homburg, Germany) during the 25 μg/day/mouse leptin infusion phase of study (days 144–151; Figures 2A and 3A–C). O_2_ and CO_2_ measurements were taken every 26 min during a 72-h period while mice were given *ad-lib* access to LFD and water. Because of possible initial stress related to transfer to the chambers, only the last 48 h (of a total on 72 h) of measurements were used to calculate total 24-h energy expenditure (TEE; expressed in kcal/day) and respiratory quotient (RQ = VCO_2_/VO_2_). Resting energy expenditure (REE in kcal/day) was defined as the lowest one hour period of energy expenditure, which coincided with the lowest one hour of total ambulatory activity during the 48-h period; this value was extrapolated to 24 h. Non-resting energy expenditure (NREE) was calculated as the difference between TEE and REE. Physical activity was measured by an infrared beam system integrated with the LabMaster system. Total activity (beam breaks) in *X*, *Y*, and *Z* axis was stored every 26 min. The system is designed to differentiate between fine motor movement (defined as a single *X* or *Y* axis beam break), ambulatory movement (defined as the simultaneous breaking of two adjacent *X* or *Y* beams), and rearing, defined as the breaking of the *Z* axis infrared beam.

### 2.4 Regain phase

Following the removal of the last mini-pump (day 155; Figures 1 and 2A), BW and food intake (FI) were measured daily for 10 days each morning (07:45 h–08:15 h) and every 2–3 days thereafter for the following 15 days when mice had *ad-lib* access to the LFD (Figure 4A–D). Food intake was measured per cage and divided by the number of mice in the cage in order to get an estimate of individual energy intake. Body composition was obtained prior to removing last mini-pump and then 5 and 41 days post excision of the last pump.

### 2.5 Diet Preference phase

Once body weights had stabilized following excision of the last mini-pump (approximately 5 weeks), mice were individually housed and allowed to acclimatize for 1 week. Both LFD and MFD were placed at the top of each cage; BW and FI were determined daily (10:00 h–11:00 h). Feed efficiency was estimated by dividing 24 h weight change by the number of calories consumed during that period (g/kcal). Following 10 days of diet preference testing, mice were given *ad-lib* access only to the MFD. Body weight was measured every 1–2 weeks during this 8-week period.

### 2.6 Serum leptin and insulin

Blood was obtained by retro-orbital bleed following a 4-h fast eight days after every mini-pump switch during the 18-week. Leptin Infusion phase as well as 5, 41, and 118 days following the last mini-pump removal. Blood was allowed to clot for 2 h at room temperature, spun at 4 °C for 20 min at 1000 × *g*, and serum was collected and frozen at −80 °C until time of assay. Leptin was assayed using the Quantikine ELISA kit (R&D Systems, Minneapolis, MN) and insulin using the Mercodia Ultrasensitive Mouse Insulin ELISA (Mercodia, Uppsala, Sweden).

### 2.7 Statistical analyses

Data are expressed as means ± SE. Statistical analyses were performed using JMP (version 7; SAS, North Carolina). Where applicable, ANCOVAs were conducted using diet group (with LEP or PBS) as independent variables and FM and FFM as covariates. Statistical significance was prospectively defined as *pα* <0.05.

## 3 Results

### 3.1 Leptin Infusion phase

#### 3.1.1 Body weight, body composition, and circulating leptin concentration

Body weight (BW) and body composition upon implantation of the first mini-pump (day 24; Figure 2A–C) were indistinguishable between LEP- and PBS-treated mice (BW: 24.0 ± 0.4 vs. 24.0 ± 0.6, fat-free mass: 17.8 ± 0.4 vs. 17.7 ± 0.4, and fat mass: 3.1 ± 0.1 vs. 3.4 ± 0.2 g, respectively). BW was significantly lower in the LEP group by the start of the 4 μg/day dose of leptin (day 44; Figure 2A) with the majority of this difference accounted for by a decrease in FM (−0.9 ± 0.1 g, representing a 30% decrease) in the LEP mice following implantation of the first mini-pump (1 μg/day; Figure 2B). The FM then stabilized in the LEP group (day 30), and FM remained unchanged throughout the rest of the experiment. FM was not statistically different at the first dose (1 μg/day; 2.2 ± 0.1 g) compared to the final dose (25 μg/day; 2.4 ± 0.3 g) in LEP mice but rose significantly in PBS mice (2.9 ± 0.2 vs. 4.6 ± 0.3 g respectively; *p* < 0.01). Fat-free mass was slightly lower in the LEP group compared to PBS group starting at the 4 μg/day dose, but reached statistical significance only at the 22 μg/day time point (Figure 2C). A repeated measures ANOVA revealed strong treatment (*p* < 0.001), time (*p* < 0.001), and time*treatment effects (*p* < 0.001). Circulating leptin concentrations were significantly higher in the LEP compared to PBS group starting at 7 μg/day (10.5 ± 1.7 vs. 4.6 ± 0.7 ng/ml respectively), and were >7.5 fold times higher at the 25 μg/day dose compared to the PBS group (47.3 ± 6.0 vs. 6.3 ± 3.0 ng/ml, respectively; Figure 2D).

#### 3.1.2 Energy expenditure, ambulatory activity, and respiratory quotient in LEP- (25 μg/day) and PBS-treated mice

Non-adjusted (for body composition) mean total 24 h energy expenditure (TEE) at 144–150 days was slightly but significantly lower in the LEP group to the PBS group (11.6 ± 0.2 vs. 12.2 ± 0.2 kcal/24 h respectively; *p* = 0.04; Table 1 & Figure 3A), a reflection of the lower resting energy expenditure (REE) in the LEP group (8.4 ± 0.1 vs. 9.1 ± 0.2 kcal/24 h; *p* = 0.01; Table 1). Whether adjusted for FM and FFM (Table 1), or FFM only (data not shown) by ANCOVA, TEE was no longer significantly different between the groups (*p* = 0.09 and *p* = 0.1, respectively) yet REE still remained significantly lower in the LEP compared to PBS mice. The higher TEE observed in the LEP mice at the beginning of the first lights off phase (Figure 3A) correlates well with increased ambulatory activity (Figure 3B). When TEE and REE were adjusted for FM and FFM using multiple regression analysis [5], TEE was nearly identical between LEP and PBS mice (data not shown). Both TEE and ambulatory movement were shifted to the left (i.e. increased earlier) in the LEP mice in the early lights off period (20:00 h–02:00 h; Figure 3A and B). Mean 24 h respiratory quotient (RQ) was similar in both groups (Table 1) and only minor, transient, differences were seen between the groups over the entire 48 h (Figure 3C).

### 3.2 Weight Regain phase

#### 3.2.1 Body weight, food intake, metabolic efficiency and circulating leptin concentrations

Following discontinuation of the mini-pumps (Day 55; Figure 2A), body weight and food intake were measured daily for the first 10 days, and then every 2–4 days for the subsequent 15 days. Body weights of the LEP mice increased at ∼0.2 g/day until reaching a plateau on day 7. Mean 7-day weight gain was 1.3 ± 0.2 g (Figure 4A), representing a 5.2 ± 0.8% increase in body weight (Figure 4B). PBS mice lost weight until day 4; mean weight loss in that period was 1.1 ± 0.2 g (Figure 4A), representing a 3.5 ± 0.7% decrease in body weight (Figure 4B). Body weights returned to levels prior to mini-pump removal in the PBS group at about day 10. Despite their initial weight gain following removal of the leptin-containing pumps, body weights of the LEP mice remained slightly but significantly lower than those of the PBS group until the end of the experiment (118 days following final mini-pump removal: LEP = 34.6 ± 0.8 g and PBS = 37.6 ± 1.3 g on last day of experiment; Figure 4A). Food intake was significantly higher in LEP than PBS animals on days 2–6 post pump removal, but after day 7 intake was similar to PBS mice (Figure 4C). Feeding efficiency, estimated by dividing the change in body weight (g) by the food intake (g) for 24 h, was significantly higher in LEP mice on days 2–6 (Figure 4d).

Four-hour fasting leptin serum concentrations were measured 5, 41, and 118 days following the cessation of leptin infusion (Table 2). LEP mice had significantly higher concentrations of leptin than PBS mice at both day 5 (75% greater) and day 41 (65% greater) post-cessation of leptin infusion, in spite of having lower fat mass (41% and 31% lower FM in LEP vs. PBS, respectively; Table 2). When adjusted for FM, the differences were further accentuated. LEP mice had 3–5 fold higher FM-adjusted circulating leptin concentrations at day 5 and 41 post-cessation of leptin administration (*p* < 0.05). Forty-two days following cessation of leptin infusion, a diet preference test (see results in next section) was started in which mice were given free access to both MFD and LFD for 10 days before being switched to MFD only. On day 118 post-cessation of leptin infusions, no significant difference in non-adjusted (for fat mass) serum leptin concentrations was observed between LEP and PBS mice, yet leptin concentrations were still significantly elevated when adjusted for fat mass using a linear regression model (LEP = 44.3 ± 4.2 ng/ml vs. PBS = 24.8 ± 6.8; *p* < 0.05). Leptin concentrations in both groups had risen significantly from the 41-day post-leptin infusion timepoint due to the increase in body fat resulting from *ad libitum* intake of the MFD.

### 3.3 Diet Preference test

Both LEP and PBS mice preferred the MFD to the LFD diet, consuming more than 90% of their entire total daily caloric intake as MFD (Figure 5). The LEP mice ingested slightly fewer total calories as a result of relatively lower intake of the MFD during the first 5 days, a trend that was reversed on the last 2 days. When EI was normalized to estimates of FM and FFM using regression analysis, there was no longer a difference in energy intake data (data not shown). Following this 10-day period, all mice were given *ad-libitum* access to the MFD.

## 4 Discussion

Leptin's role in signaling peripheral energy stores (i.e. adiposity) to the CNS has been well documented [21]. Whether decreased CNS sensitivity to leptin is a cause or a consequence of obesity is unclear [19]. These possibilities are, of course, not mutually exclusive. *Lep*^*ob*^ mice, whose circulating leptin concentrations are matched to those of lean animals by low dose administration of leptin via mini-pump, remain leptin sensitive (as manifest by hypothalamic phospho-stat3 response) even after becoming obese in response to feeding of a high fat diet for 20 weeks [22]. In these experiments, *Lep*^*ob*^ mice were administered exogenous leptin sufficient to achieve circulating leptin concentrations that were comparable to WT mice fed a low fat diet. WT and leptin-supplemented *Lep*^*ob*^ mice were then fed a high fat diet *ad-libitum* for 20 weeks and allowed to get obese. The WT mice that were switched to the HFD became leptin resistant (decreased pSTAT3 activation in the hypothalamus following leptin administration), but the leptin-supplemented *Lep*^*ob*^ mice, which became comparably fat, were not leptin resistant, suggesting that hyperleptinemia *per se* is required to induce the leptin resistance associated with obesity [22]. The use of a high fat diet in this context also suggests that hypothalamic leptin resistance is not a consequence of the HFD *per se*, but requires an interaction of hyperleptinemia with metabolic consequences of the HFD [22]. We have shown that elevation of body weight (and consequently circulating leptin concentrations) for a period of 16 weeks in C57BL/6J mice results in metabolic defense of the higher body weight as manifest in reduced energy expenditure normalized to body mass/composition following weight reduction [5]. The goal of the present study was to determine whether 18 weeks of hyperleptinemia (without the metabolic/neurobiologic “confound” of obesity) would likewise invoke an upward resetting of defended body weight in mice fed a low (10%) fat diet. We found that chronic elevations of circulating leptin concentrations, *per se*, did not lead to metabolic or behavioral “defense” of a higher body weight.

Leptin infusion resulted in lower fat mass accumulation starting at infusion rates of 4 μg/day in LEP- compared to PBS-infused control mice (fat mass = 1.9 ± 0.1 vs. 3.0 ± 0.2 g respectively; *p* < 0.05). Harris et al. showed that *Lep*^*ob*^ mice receiving peripheral doses of 2 μg/day via osmotic mini-pump significantly decreased food intake and body weight, while a dose of 10 μg/day was required to produce similar changes in wild type mice [23]. In a separate study, 200 ng/h infusion rates (i.e. 4.8 μg/day) significantly decreased body weights of wild type mice by approximately 5% over a 14-day infusion period; there was a positive correlation between leptin infusion rates (200, 300, 400, and 500 ng/h) and maximal weight loss [24]. The highest dose administered in the present study (25 μg/day = 1042 ng/h) is higher than the 10 μg/day that was reported to greatly reduce somatic fat in mice [10]. 25 μg/day in the present study did not decrease the RQ of LEP mice compared to PBS mice (0.88 ± 0.01 vs. 0.89 ± 0.01 respectively) suggesting that a gradual increase in circulating leptin results in decreased sensitivity to leptin's effects on fat catabolism [25]. When the leptin infusion was stopped, LEP-infused mice fed a low fat diet had a 5.2 ± 0.8% weight increase during the first 10 days (vs. −1.0 ± 0.8% in the PBS mice) and then stabilized at a body weight slightly (≈5%) but significantly lower than the PBS-infused mice, suggesting that the “set point” for body fat had not been reset upwards, and may even have been reduced. Weight regain following a period of exogenous intracerebroventricular leptin administration (at a dose of 8 ng/h) has been well documented in mice, and may result from a “perceived” leptin deficiency in the CNS following the discontinuation of leptin infusion [24]. However, since fat mass is reduced as a result of leptin infusion, other adipose tissue-mediated signals cannot be ruled out as a cause of weight regain following infusion cessation [23,24]. Acute reduction in circulating leptin concentration should result in suppressed metabolic rate following removal of the pump and, although not directly measured by indirect calorimetry in the present study, the high feed efficiency may reflect, in part, a lower metabolic rate. The relative decline in REE (normalized for body mass and composition) observed just prior to cessation of the LEP infusion is of some interest with regard to our initial hypothesis. The LEP infusion itself would be expected to slightly increase or have no effect on the energy expenditure of these animals [26,27]. The decrease in REE noted might be evidence of a subtle upward resetting of the “adipostat” reflecting a homeostatic decline in energy expenditure that would favor an increase in body fat content. Although plausible, this formulation seems unlikely since cessation of the leptin infusion was followed by only a small increase in body weight. A related mechanistic possibility is that anti-LEP antibodies may have impaired LEP bioavailability in the CNS. Again, the modest and apparently limited impact of the decline in REE is against this interpretation. Finally, the slight decline in REE may reflect a change in some other metabolite/adipokine in the LEP-treated animals. A lower metabolic rate coupled with increased food intake would synergize to quickly increase fat mass as observed in the present study (Figures 3A and 4C/D). At the end of the leptin infusion phase, LEP mice had nearly 8-fold greater circulating leptin concentrations than PBS mice (47.3 ± 6.1 vs. 6.2 ± 1.1 ng/ml respectively), and lower absolute (2.4 ± 0.3 vs. 4.6 ± 0.3 g, respectively) and percent (7.7 ± 1.0% vs. 12.7 ± 1.4%, respectively) fat mass. The leptin concentrations observed in the LEP mice are commensurate with those found in HFD-fed mice at 16 weeks of age (10 weeks of high fat diet feeding). Once mini-pumps were removed, leptin concentrations in the LEP mice fell by >50% over 5 days, dropping from 47.3 ± 6.1 ng/ml during the 25 μg/day infusion period to 20.5 ± 3.4 ng/ml. However, the serum leptin concentrations of LEP mice remained significantly higher than those of the PBS-treated mice, despite the significantly lower fat mass (≈40% lower; LEP = 2.4 ± 0.3 vs. PBS = 4.4 ± 0.4 g) of the LEP mice. LEP mice display increased circulating leptin concentrations adjusted for FM even 141 days following mini-pump removal. These apparently elevated circulating leptin concentrations may reflect several possible (not mutually exclusive) mechanisms: 1) leptin production per unit FM is actually elevated but has limited influence on metabolic parameters and/or development or persistence of induced hypothalamic leptin resistance; 2) reduced clearance or catabolism of leptin; 3) increase in the soluble leptin receptor (LepR) decreasing the bioavailability of leptin binding to LepRb, thereby resulting in increased leptin production; 4) the chronic leptin infusions may have stimulated the production of leptin antibodies resulting in an artifactual increase in apparent serum leptin concentration in the ELISA assay [28,29]; and/or 5) the mice developed neutralizing antibodies to exogenous leptin, as has been reported in a small number of humans receiving prolonged exogenous leptin therapy [30].

Seventy-one percent of human subjects to whom leptin was administered in high doses for 24 weeks developed non-neutralizing anti-leptin antibodies [11]. Insufficient serum was available in our LEP mice to assess the presence of anti-leptin antibodies. Titers of neutralizing antibodies for leptin found in human patients [30] were inversely correlated with weight loss [31]. Mice and rats lacking functional alleles of the leptin receptor (*Lepr*^*db/db*^ and *Lepr*^*fa/fa*^, respectively) have higher circulating leptin concentrations per unit of fat mass than +/+ controls [32,33]. Rodents heterozygous for the *Lepr* mutation are fatter than +/+ animals and consequently have increased plasma leptin concentrations which are, however, comparable per unit fat mass [33,34]. Normalized (to FM) plasma leptin concentrations in 10-day old *Lepr*^*fa/fa*^ or *Lepr*^*fa/+*^ rat pups are 3.5 and 1.6 times higher, respectively, than in *Lepr*^*+/+*^ animals, and these increases are associated with increased leptin mRNA levels in adipose tissue (60% higher in *Lepr*^*fa/fa*^ and 26% higher in *Lepr*^*fa/+*^ when compared to *Lepr*^*+/+*^ animals) [33]. *Lepr*^*fa/fa*^ rats clear leptin from the circulation at a lower rate due presumably to leptin binding to its soluble receptor which is increased 20-fold in the plasma of *Lepr*^*fa/fa*^ rats [35].

Leptin decreases responses related to food reward through actions mediated by the midbrain dopamine and opioidergic pathways, effects that may result in alterations in food preference [36,37]. Two months following discontinuation of the leptin infusion, a diet preference test showed no differences between LEP and PBS mice when offered both 10% and 30% fat diets *ad-libitum* (Figure 5). Weight-reduced humans show changes in brain region-specific neural activity compared to themselves prior to weight loss. Many of these changes are reversed by “replacement” doses of leptin, in brain areas known to be involved in the regulatory, emotional, and cognitive control of food intake, further suggesting a link between leptin and cognitive food cues [38].

The results in this manuscript suggest that chronic elevations of circulating leptin concentrations *per se* do not result in major changes in defended body weight or diet preference. Peripheral leptin injection/infusion studies in mice have focused on the effects of leptin on body weight and composition, glucose homeostasis, and leptin resistance in the central nervous system. Infusion periods have ranged from single injections, to 28-day studies at infusion rates from 1 μg/day to 40 μg/day [10,23,24,39]. Our study was designed to examine directly the effects of step-wise increases in circulating leptin concentrations (in the absence of a HFD) over a period of 18 weeks on body weight regulation, and whether such elevations would produce a permanent elevation in the level of defended body weight (fat). The design explicitly isolated the effects of elevated leptin *per se*, from the effects of increased somatic and dietary fat. We have previously suggested [5] that the hypometabolic phenotype observed in DIO mice that are weight-reduced to 20% below their “normal” body weight may result from hypothalamic structural changes that result in perceived relative “hypoleptinemia”. Chronic hyperleptinemia could affect both molecular and structural substrates for response to leptin in the central nervous system and elsewhere [5]. The fact that LEP mice in this study did not “defend” an elevated body weight following the discontinuation of the leptin infusion suggests that 1. the period of leptin infusion was not sufficiently long; 2. diet/adiposity-mediated (rather than elevated leptin *per se*) changes in the CNS may underlie the defense of an elevated body weight [5]; 3. Elevated leptin concentrations and factors related to #2 act synergistically to produce an elevation in the set point or threshold of defended body fat; and/or 4. Antibody production against exogenous leptin may have reduced bioavailability for mediating the processes of structural change that would presumably underlie increased threshold in the CNS. When administered by either CNS or peripheral infusion, leptin increases energy expenditure [40]. The 5% lower unadjusted (for body weight/composition) energy expenditure observed in the LEP mice receiving 25 μg/day leptin (*p* = 0.04), suggests that slow increases in ambient leptin by infusion may lead to desensitization to some of the metabolic and behavioral effects of acute leptin administration.

## Figures and Tables

**Table 1:** Non-adjusted and adjusted (using ANCOVA with FM & FFM as covariates) total energy expenditure (TEE), resting energy expenditure (REE), and non-resting energy expenditure of LEP and PBS mice during last mini-pump infusion (25 μg/day of leptin).

	Energy expenditure			
	TEE (kcal/24 h)	REE (kcal/24 h)	NREE (kcal/24 h)	RQ (24 h)
Non-adjusted				
PBS (*n* = 12)	12.2 ± 0.2	9.1 ± 0.2	3.2 ± 0.1	0.89 ± 0.01
LEP (*n* = 32)	11.6 ± 0.2*	8.4 ± 0.1*	3.2 ± 0.1	0.88 ± 0.01

Adjusted (FM & FFM)				
PBS (*n* = 12)	12.1 ± 0.2	9.1 ± 0.3	3.2 ± 0.1	0.89 ± 0.01
LEP (*n* = 32)	11.8 ± 0.3	8.5 ± 0.1^#^	3.2 ± 0.1	0.88 ± 0.01

* Significantly different between groups by *t*-test (*p* < 0.05). ^#^ Significantly different between groups by ANCOVA treating FM & FFM as covariates (*p* < 0.05).

**Table 2:** Non-adjusted and adjusted (using ANCOVA with FM as covariate) leptin concentrations (ng/ml) at 3 time points (5, 41, and 118 days) following cessation of leptin infusion.

	Serum leptin concentration (ng/ml)		
	Leptin (5 days)	Leptin (41 days)	Leptin (118 days)
Non-adjusted			
PBS (*n* = 12)	11.7 ± 2.7	10.5 ± 2.3	34.6 ± 5.1
LEP (*n* = 32)	20.5 ± 3.4*	17.3 ± 3.7*	40.3 ± 6.3

Adjusted mean (FM)			
PBS (*n* = 12)	4.7 ± 5.1	5.7 ± 5.4	24.8 ± 6.8
LEP (*n* = 32)	23.1 ± 4.7^#^	19.1 ± 3.2^#^	44.3 ± 4.2^#^

* Significantly different between groups by *t*-test (*p* < 0.05). ^#^ Significantly different between groups by ANCOVA treating FM as covariate (*p* < 0.05).
